# Effects of Subthalamic Nucleus Lesions and Stimulation upon Corticostriatal Afferents in the 6-Hydroxydopamine-Lesioned Rat

**DOI:** 10.1371/journal.pone.0032919

**Published:** 2012-03-12

**Authors:** Ruth H. Walker, Cindy Moore, Georgia Davies, Lisa B. Dirling, Rick J. Koch, Charles K. Meshul

**Affiliations:** 1 Department of Neurology, James J. Peters Veterans Affairs Medical Center, Bronx, New York, United States of America; 2 Department of Neurology, Mount Sinai School of Medicine, New York, New York, United States of America; 3 Research Services, Neurocytology Laboratory, Veterans Affairs Medical Center, Portland, Oregon, United States of America; 4 Department of Behavioral Neuroscience and Pathology, Oregon Health & Science University, Portland, Oregon, United States of America; The Mental Health Research Institute of Victoria-The University of Melbourne, Australia

## Abstract

Abnormalities of striatal glutamate neurotransmission may play a role in the pathophysiology of Parkinson's disease and may respond to neurosurgical interventions, specifically stimulation or lesioning of the subthalamic nucleus (STN). The major glutamatergic afferent pathways to the striatum are from the cortex and thalamus, and are thus likely to be sources of striatal neuronally-released glutamate. Corticostriatal terminals can be distinguished within the striatum at the electron microscopic level as their synaptic vesicles contain the vesicular glutamate transporter, VGLUT1. The majority of terminals which are immunolabeled for glutamate but are not VGLUT1 positive are likely to be thalamostriatal afferents. We compared the effects of short term, high frequency, STN stimulation and lesioning in 6-hydroxydopamine (6OHDA)-lesioned rats upon striatal terminals immunolabeled for both presynaptic glutamate and VGLUT1. 6OHDA lesions resulted in a small but significant increase in the proportions of VGLUT1-labeled terminals making synapses on dendritic shafts rather than spines. STN stimulation for one hour, but not STN lesions, increased the proportion of synapses upon spines. The density of presynaptic glutamate immuno-gold labeling was unchanged in both VGLUT1-labeled and -unlabeled terminals in 6OHDA-lesioned rats compared to controls. Rats with 6OHDA lesions+STN stimulation showed a decrease in nerve terminal glutamate immuno-gold labeling in both VGLUT1-labeled and -unlabeled terminals. STN lesions resulted in a significant decrease in the density of presynaptic immuno-gold-labeled glutamate only in VGLUT1-labeled terminals. STN interventions may achieve at least part of their therapeutic effect in PD by normalizing the location of corticostriatal glutamatergic terminals and by altering striatal glutamatergic neurotransmission.

## Introduction

Deep brain stimulation (DBS) and lesioning of basal ganglia targets have become state-of-the-art therapies for selected patients with moderately advanced Parkinson's disease (PD). However, the mechanisms by which neurosurgical interventions improve the symptoms of PD are not well understood - whether clinical benefit is achieved by neuronal excitation, inhibition, modulation, or some combination of these. Stimulation and lesioning have similar, but not identical, clinical effects. The effects of lesioning are by definition inhibitory, as neuronal structures are destroyed, but there is evidence to support both inhibitory and excitatory mechanisms of stimulation at the neuronal level [1], [2].

Using microdialysis we have previously found changes in striatal extracellular glutamate levels in rats which had undergone 6-hydroxydopamine (6OHDA) lesions, subthalamic nucleus (STN) lesions and STN stimulation [3]. The relative contributions of the different glutamatergic inputs to the striatum in the setting of dopamine depletion are not well understood, but have implications, for example, for the possible manipulation of a particular pathway or neurotransmitter for therapeutic ends. In order to determine the neuronal source of these changes in glutamate, and to provide insights into alterations in basal ganglia functioning in this situation we performed electron microscopic (EM) studies using immuno-gold to label glutamate [4] and antibodies against the glutamate transporter VGLUT1 to identify corticostriatal afferents [5]–[8].

We focused upon VGLUT1-immunolabeled glutamatergic, presumptively corticostriatal, synapses [5]–[8], in order to study changes to this pathway in the setting of dopamine depletion and of surgical manipulations of the STN. The other major glutamatergic input to striatum from the thalamic centromedian parafascicular (CM-pf) nucleus contains only VGLUT2. Although VGLUT1 mRNA is present in the thalamic motor relay nuclei [9], [10], which have a lesser, although still significant striatal input, immunocytochemical studies suggest that VGLUT1 is found only in corticostriatal terminals [6], [7], [11]–[13]. VGLUT1 and VGLUT2 afferents both provide similar innervation to D1 and D2-receptor-bearing striatal neurons [14].

We hypothesized that corticostriatal and non-corticostriatal terminals would be differentially affected in the different experimental groups, providing information about the neuronal source of changes in striatal glutamate in the dopamine-depleted state and following STN surgical interventions. We studied the localization of VGLUT1-labeled synaptic terminals in 6OHDA-lesioned rats, and following short term, high frequency STN stimulation and STN lesions. The localization of glutamatergic inputs upon heads or necks of spines, or upon the shafts of dendrites of striatal medium spiny neurons is likely to play a critical role in the integration and processing of incoming neuronal signals [15]. Dopaminergic and thalamic inputs located upon the shafts of dendrites may have a general modulatory effect upon signals from glutamatergic inputs on spine heads [15]. We also examined effects of STN manipulations in 6OHDA-lesioned rats upon pre- and post-synaptic glutamate immunolabeling in VGLUT1-labeled vs. -unlabeled terminals.

## Materials and Methods

### Ethics statement

All animal procedures were approved by the Institutional Animal Care and Use Committee of the James J. Peters Veterans Affairs Medical Center (protocol #6531-04-020).

### Experimental groups

Young adult (200–250 g) male Sprague-Dawley rats were used. Five groups of rats were used for EM studies; controls (n = 8), 6OHDA lesions alone (n = 6), 6OHDA+ STN electrolytic lesions (n = 5), and 6OHDA+sham (n = 3) or active (n = 4) STN stimulation.

### 6-hydroxydopamine lesions

**Figure 1:**
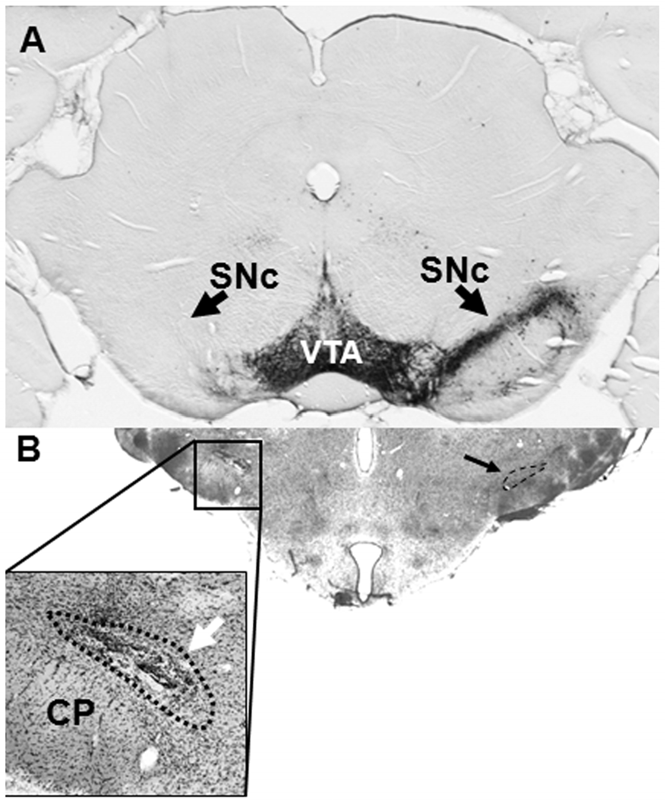
Nigral and subthalamic lesions. (A) Depletion of TH-immunoreactive neurons in left substantia nigra pars compacta, as compared with the unlesioned right side (arrows). (B) Electrolytic ablation of left subthalamic nucleus (white arrow; outlined by black dotted line); results were only analyzed from animals in which at least 50% of the subthalamic nucleus was lesioned; the intact contralateral subthalamic nucleus is indicated on the right side (small black arrow; dashed outline). CP = cerebral peduncle, SNc = substantia nigra, pars compacta, VTA = ventral tegmental area.

After acclimation, animals were anaesthetized with intraperitoneal injections of ketamine (40 mg/kg) and xylazine (5 mg/kg), and placed in a stereotaxic frame (Kopf, Tujunga, CA). Two injections of 12 µg 6OHDA (Sigma; 2 µl injections of 6 mg/ml in 0.9 M NaCL, with 0.2 mg/ml L-ascorbate on ice) were performed as previously reported [3], [16], targeting the substantia nigra, pars compacta (SNc), at 2 sites, 1.8 mm lateral and 5.6 mm posterior to bregma, and 1.6 mm lateral and 5.8 mm posterior, with the aim of producing a relatively complete lesion. 10–14 days later, rats were injected intraperitoneally with amphetamine (2.5 mg/kg). Only rats which turned >50 turns/10 minutes were used for subsequent procedures. The extent of the lesion was confirmed with immunohistochemistry against tyrosine hydroxylase (TH) [3], [16], [17] (Fig. 1A).

### Subthalamic nucleus lesions

Two weeks after 6OHDA lesions, one group of rats (n = 5) underwent electrolytic ablation of the ipsilateral STN. This was performed using a monopolar lesioning electrode (Fred Haer & Co. Inc., Bowdoinham, ME) at 2 sites (2.3 mm lateral, 3.4 and 3.6 mm posterior to bregma, 7.9 mm ventral to dura) [3], [16]. Following perfusion of the animals with fixative (see *Electron microscopy*), the brains were serially cut at 60 µm through the STN using a vibratome (Ted Pella, Redding, CA). All sections through the lesioned STN were mounted on glass slides and stained with thionin. The area of the STN was determined on serial sections and the percent of the STN which had been lesioned was determined on each section for each animal. Results were only analyzed from animals in which at least 50% of the STN was lesioned and there was no significant damage to the surrounding tissue (Fig. 1B).

### STN stimulating electrode placement and stimulation

For STN stimulation, two weeks after 6OHDA lesions, a stainless steel, bipolar, concentric stimulating microelectrode (tip diameter 200 µm; FHC) was implanted into the ipsilateral STN (2.3 mm lateral, 3.6 mm posterior to bregma, 7.9 mm ventral to dura) [3], [16].

One week later, for animals undergoing active stimulation (n = 4), the implanted stimulating electrode was connected to a rectangular pulse generator (Pulsar Digital Stimulator 6-bp, FHC) with the inner pole negative, and stimulation was carried out at 300 µA at 130 Hz [3], [16], [18], [19] for one hour prior to euthanasia. No involuntary movements were noted during stimulation. A parallel group of animals (n = 3) had electrodes placed but did not undergo stimulation (sham stimulation). Following perfusion with fixative, brains were serially sectioned through the STN and mounted for confirmation of the location of the electrode track. Only those animals in which the electrode track was found within the STN were used for the final analysis.

### Electron microscopy

Animals were euthanized for EM one week after STN surgery with a lethal dose of anesthetic (ketamine 80 mg/kg, xylazine 10 mg/kg). The chest cavity was opened for transcardial perfusion with 6 ml of heparin (1000 units/ml) in 0.1 M HEPES buffer pH 7.3, followed by 300 mls of 2% paraformaldehyde/0.1% glutaraldehyde/0.1% picric acid in the same buffer, all at room temperature. All animals from each of the experimental groups were perfused with fixative on the same day in order to limit the variables that may occur by perfusing the rats on different days. This avoided any potential problems due to brain anoxia or tissue perfusion that would influence the glutamate immuno-gold labeling results.

For EM immunolabeling for VGLUT1 and glutamate immuno-gold labeling the rostral block of brain tissue was sectioned on the vibratome at 60 µm, and the dorsolateral striatum dissected. This is equivalent to +1.0 mm anterior [20], which receives a glutamatergic projection from the somatosensory cortex [21] and is involved in the motor circuit.

Striatal tissue was processed for EM pre-embedding diaminobenzidine (DAB) immunolabeling [VGLUT1 (1∶10,000), rabbit polyclonal, Synaptic Systems, Germany] as previously described [22], with modifications below using a new microwave procedure [23]. Tissue was incubated in the microwave (Pelco BioWave, Ted Pella, Inc) for 5 minutes, 550 watts (W), at 35°C with the vacuum off (all the remaining steps occurred at this temperature) in 10 mM sodium citrate, pH 6.0, rinsed in 0.1 M phosphate buffer (PB) for 2×1 min at 150 W, with the vacuum off, exposed to 3% hydrogen peroxide at 150 W for 1 min with the vacuum on, rinsed in PB at 150 W for 2×1 min with the vacuum off, exposed to 0.5% Triton X-100 for 5 min, 550 W with the vacuum on, washed in PB for 2×1 min at 200 W with the vacuum off, then exposed to the primary antibody for 12 minutes at 200 W, 4 times using the following cycle: 2 min on, 2 min off, 2 min on, 5 min off, all on a continuous vacuum. The tissue is then rinsed in PB, 2×1 min, at 150 W with the vacuum off, then exposed to the secondary antibody (biotinylated goat anti-rabbit, 1∶500; Vector Labs) for 15 minutes at 200 W for two cycles of the following: 4 min on, 3 min off, 4 min on, 5 min off, all on a continuous vacuum. The tissue is then rinsed in PB, 2×1 min, at 150 W with the vacuum off, then exposed to ABC (Vector Elite Kit, 1 µl/ml of solution A and B in PB) for 11 minutes at 150 W, under vacuum, using the following cycle: 4 min on, 3 min off, 4 min on. The tissue is then rinsed in PB, 2×1 min, at 150 W with the vacuum off and then exposed to DAB (0.5 µg/ml+1.5% hydrogen peroxide) for up to 10 minutes at room temperature.

The tissue was embedded in epoxy for immuno-gold labeling as previously described [4]. All tissue from each of the experimental groups was cut and processed on the same day in order to limit the variables that may occur by cutting and processing tissue on different days.

Post-embedding immuno-gold electron microscopy was performed [24] with modifications [4]. The glutamate antibody (non-affinity purified, rabbit polyclonal; Sigma Chemical Co., St. Louis, MO), as previously characterized [24], was diluted 1∶250 in TBST 7.6. Aspartate (1 mM) was added to the glutamate antibody mixture 24 hours prior to incubation with the thin-sectioned tissue to prevent any cross-reactivity with aspartate within the tissue. The secondary antibody was goat anti-rabbit IgG (Amersham, Arlington Heights, IL; diluted 1∶25 in TBST pH 8.2), tagged with 10 nm gold particles. We previously reported that incubation of the antibody with 3 mM glutamate resulted in no immuno-gold labeling, showing the specificity of the glutamate labeling [25]. Photographs (10/animal) of VGLUT1-labeled and -unlabeled terminals were taken from a single 50 µm mesh grid (1 thin section/grid, 1 photograph/grid square) throughout the neuropil (an area containing the highest numbers of synapses) at a final magnification of ×40,000 by an individual blinded to the experimental groups, using a digital camera (AMT, Danvers, MA). Photographs were randomly taken throughout the neuropil, the area containing the highest number of synapses, but only in areas where there was VGLUT1/DAB reaction product. Such labeled structures formed the lowest part of the photograph in order to ensure that VGLUT-1-unlabeled terminals were not counted if they were found lower than the deepest VGLUT1-labeled structures. VGLUT1/DAB-labeled or -unlabeled terminals were analyzed only if there was an accumulation of synaptic vesicles within the nerve terminal and the terminal was seen making an asymmetrical synaptic contact with an underlying dendrite or dendritic spine.

For quantification of glutamate labeling, the number of immuno-gold particles located either within, or at least touching the synaptic vesicle membrane (i.e. vesicular pool), the number located outside the synaptic vesicles (i.e. the cytoplasmic pool, which is most likely the origin of the glutamate for the cystine-glutamate antiporter), and those associated with mitochondria, were counted. The vesicular and cytoplasmic pools were combined since the cytoplasmic pool is very small (<10%) compared to the vesicular pool [4]. We have reported that nerve terminals making a symmetrical contact contain GABA [4], the precursor for which is glutamate. Therefore nerve terminals making a symmetrical contact will naturally contain some glutamate immunolabeling and cannot be considered immuno-negative as a way of determining a ratio between glutamatergic and GABA-ergic terminals [4], [26]. The metabolic pool is also relatively small and thus unlikely to be a major source of variation in labeling intensity. In the current study, we find no differences in the density of mitochondrial glutamate immuno-gold labeling between the control compared to all other treatment groups in either the VGLUT1-labeled or -unlabeled terminals (data not shown).

**Figure 2:**
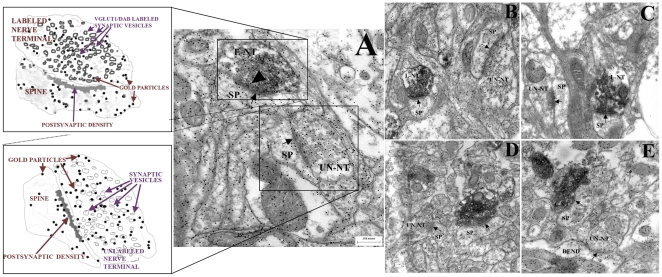
Immuno-gold labeled glutamate particles in VGLUT1-labeled and -unlabeled terminals. (A) **Control**. Electron photomicrographs of VGLUT1-labeled (L-NT) and -unlabeled (UN-NT) nerve terminals making asymmetric synaptic contacts (arrow) onto dendritic spines (SP). The 10 nm gold particles within the nerve terminals (see arrowhead in the L-NT) indicate the location of an antibody against glutamate. The inserts show labeled details of a terminal containing VGLUT1-immunoreactive synaptic vesicles (above) and a terminal containing unlabeled synaptic vesicles (below). (B) **6-OHDA lesion**. L-NT and UN-NT both make asymmetric synaptic contacts (arrows) with SPs. The density of immuno-gold labeling within these terminals is similar to that of the control group (A). (C) **6-OHDA/STN lesion**: L-NT and UN-NT make asymmetric synaptic contacts (arrows) with SPs. The density of nerve terminal immuno-gold labeling is significantly reduced compared to the control (A) and 6OHDA (B) tissue. (D) **6-OHDA/STN stimulation**. Both L-NT and UN-NT make asymmetrical synaptic contacts (arrows) with SPs. The density of nerve terminal glutamate immuno-gold labeling is similar to that seen in the 6-OHDA/STN lesion group (C). (E) **6-OHDA/Sham-Stim**. The L-NT makes an asymmetric synaptic contact with the SP, and the UN-NT makes an asymmetric synaptic contact with a dendritic shaft (DEND). The density of nerve terminal glutamate immuno-gold labeling is greater than in the 6-OHDA/STN lesion (C) and 6-OHDA/STN stimulation (D) tissue. Calibration bar: 0.25 microns.

For all VGLUT1-immunolabeled terminals and non-VGLUT1-labeled terminals (Fig. 2) we measured the density of immuno-gold labeling in each terminal, to determine the effects of the lesions upon each class (i.e. corticostriatal versus non-corticostriatal) of striatal glutamatergic afferents. The density of gold particles/µm^2^ of nerve terminal area for the vesicular/cytoplasmic and metabolic pools was determined for each animal and the mean density for each treatment group calculated. Background labeling was determined within glial cell processes and was found to be 10 immuno-gold-labeled particles/µm^2^ [25]. This was subtracted from the density of presynaptic immuno-gold-labeled glutamate glutamate within the nerve terminals for all treatment groups. We also examined the density of immuno-gold-labeling in post-synaptic spines, as changes in post-synaptic glutamate may reflect alterations in synaptic metabolic activity. In addition, since the glutamate transporter, EAAC1 (excitatory amino acid carrier 1) is neuronal and located post-synaptically, changes in glutamate density within the spine may also reflect alterations in neuronal uptake of glutamate.

In order to obtain an estimate of whether there was any change in number of glutamatergic synapses following dopamine depletion, we counted the number of synapses in each photographic field of view (32 µm^2^) upon either spines or dendritic shafts, made by either VGLUT1-labeled or –unlabeled terminals. In addition, the percentage of VGLUT1-labeled or -unlabeled nerve terminals making an asymmetrical synaptic contact onto a dendrite or dendritic spine was calculated. The same nerve terminals that were part of the analysis for the density of glutamate immuno-gold labeling (see above) were also used to determine what percentage were contacting either a dendrite or spine.

The analysis of the treatment groups was carried out in a blinded fashion. The differences between treatment groups were analyzed with a one-way ANOVA and significant main effects were further characterized using the Fisher post hoc test for comparison of multiple means.

## Results

### Unaffected parameters

In none of the groups did we see a significant change in the proportions of terminals at asymmetric synapses which were VGLUT1-labeled (approximately 55%) as opposed to -unlabeled (approximately 45%) (data not shown). This percentage is larger than previously reported in the rat [6] (34.8%) but was similar to that in the monkey (51.9%) [7]. In the MPTP-treated primate there was a relative increase in VGLUT1-labeled terminals [7], which may be at least in part attributable to differences in time course and methodology.

In estimating the numbers of synapses/32 µm^2^ made by glutamatergic terminals upon spines or shafts, we did not find statistically significant differences between the experimental groups (Table 1). There was, however, a decrease of 18.4% in VGLUT1-labeled terminals and a decrease of 18.7% in non-VGLUT1-labeled terminals making an asymmetrical synaptic contact between controls and 6OHDA-lesioned rats. The total number of all glutamatergic synapses/32 µm^2^ decreased by 18.5% between the controls and the 6OHDA group, similar to the value reported using formal stereology (19%) [27]. There was no change between groups in cross-sectional area of terminals (data not shown).

**Table 1:** Mean numbers of VGLUT1-labeled or –unlabeled synapses on spines and shafts per field of view (32 µm^2^) ± standard deviation. Differences were not statistically significant.

Group	VGLUT1-labeled		Non-VGLUT1-labeled	
	On spines	On shafts	On spines	On shafts
Control	1.75±0.39	0.15±0.12	1.32±0.27	0.25±0.14
6OHDA	1.31±0.24	0.28±0.16	1.06±0.24	0.30±0.17
6OHDA+STN lesion	1.33±0.23	0.31±0.16	1.11±0.17	0.23±0.16
6OHDA+STN stimulation	1.48±0.27	0.09±0.08	1.18±0.28	0.25±0.12
6OHDA+sham stimulation	1.9±0.61	0.36±0.17	1.37±0.15	0.33±0.12

### Localization of glutamatergic synapses in the control group

**Figure 3:**
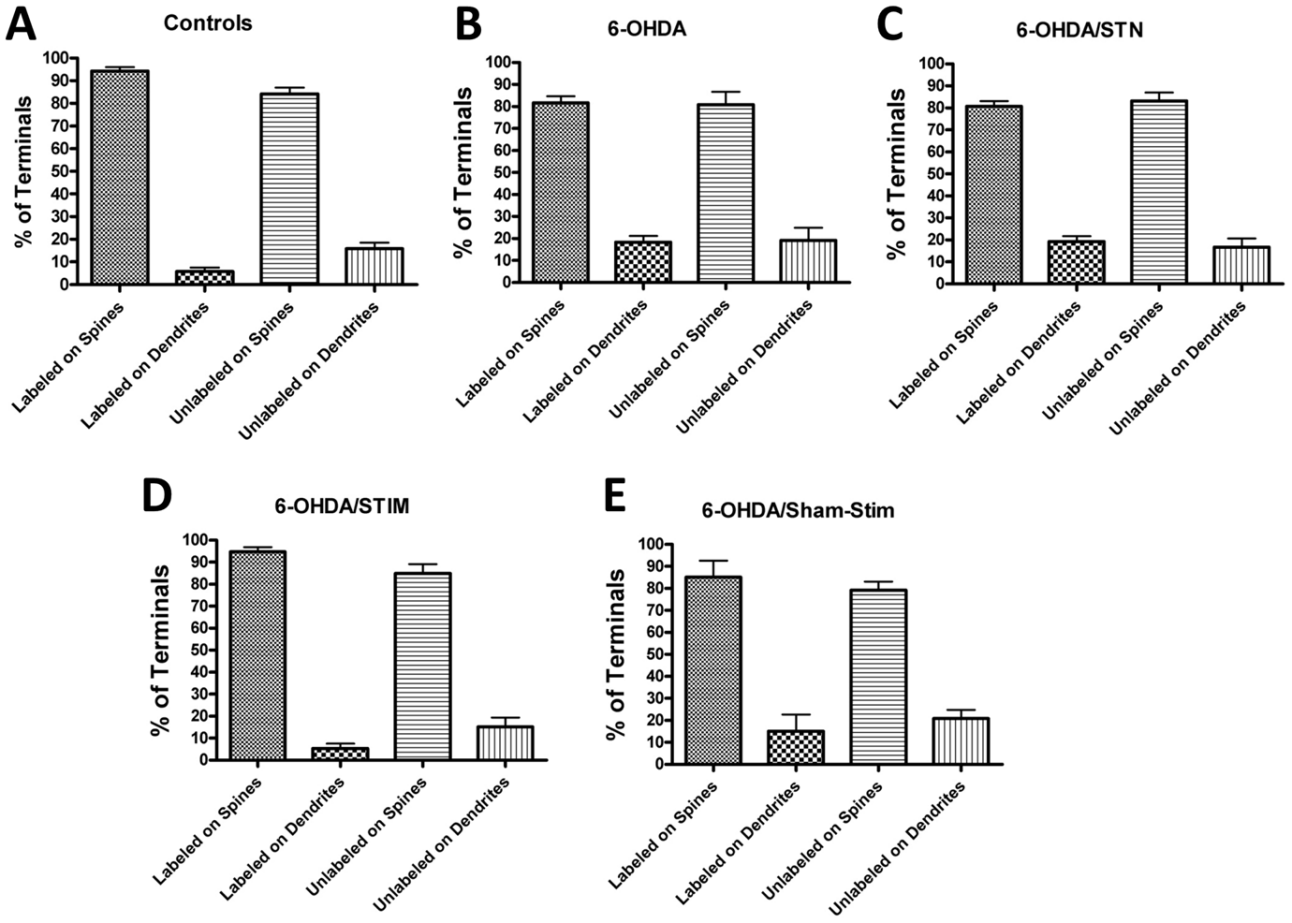
Location of VGLUT1-labeled and -unlabeled terminals for each experimental group. (A) control (n = 8), (B) 6OHDA-lesioned (n = 6), (C) 6OHDA+STN lesion (n = 5), (D) 6OHDA+STN stimulation (n = 4), (E) 6OHDA+ sham stimulation (electrode placement alone; n = 3).

**Figure 4:**
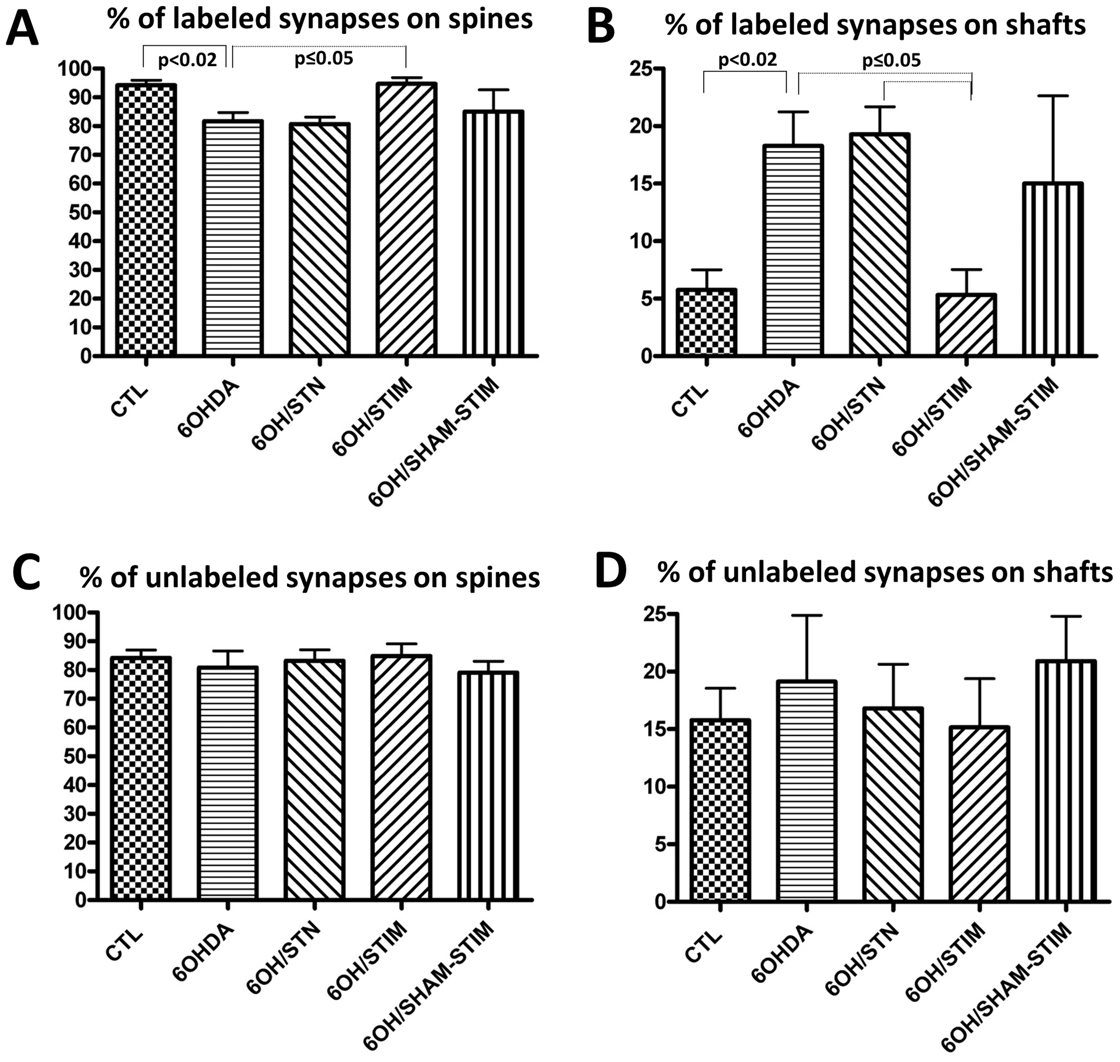
Comparison of localization of VGLUT1-labeled and -unlabeled terminals between groups. Percentages of VGLUT1-labeled terminals synapsing upon dendritic spines (A) and shafts (B) in the 5 experimental groups; control (n = 8), 6OHDA-lesioned (n = 6), 6OHDA+STN lesion (n = 5), 6OHDA+STN stimulation (n = 4), and 6OHDA+ sham stimulation (electrode placement alone; n = 3). In 6OHDA-lesioned animals, there was a shift from synapses on spines to upon shafts (p<0.02), which was reversed by STN stimulation (p<0.05). Percentages of non-VGLUT1-labeled terminals synapsing upon dendritic spines (C) and shafts (D). There were no significant differences between any of the experimental groups for unlabeled terminals.

We present the same data organized by experimental group (Fig. 3) and by type of terminals (Fig. 4), in order to compare groups. Of all VGLUT1-labeled glutamatergic (asymmetric) synapses in the control group, 94% made synaptic contact onto spines (Fig. 2A), similar to previous studies [6], [7], [28]. The remaining 6% made contact on dendritic shafts (Fig. 3A). For non-VGLUT1- labeled terminals, 84% synapsed onto dendritic spines (Fig. 2A), and the remaining 16% contacted dendritic shafts (Fig. 3A). This figure is higher than that reported by others for the rodent (72%) [6] and monkey (approximately 55%) [7]. However, it is within the range (60–85% in three rats) of specifically VGLUT2-labeled terminals making synapses upon spines [28]. In the current study, VGLUT1-unlabeled terminals account for both VGLUT2 terminals and asymmetrical contacts that are neither VGLUT1- nor VGLUT2-positive. Nearly 30% of the nerve terminals making asymmetrical synaptic contacts do not label for either VGLUT1 or VGLUT2 protein [6], thus we are unable to draw firm conclusions about the identity of unlabeled terminals in this study.

### 6OHDA lesions

Based upon our previously-published studies [3], [16], rats which rotated with amphetamine >50 turns/10 minutes had a mean depletion of 90% of TH immunoreactivity of the substantia nigra by optical densitometry as compared with the unlesioned side (Fig. 1A).

Examining only VGLUT1-labeled terminals following dopamine depletion (Fig. 3B), there was a significant decrease in the percentage that made contact on spines compared to the control group, from 94% to 82% (p<0.02; Fig. 4A). The percentage of VGLUT1- labeled terminals making contact on shafts increased from 6% in the control group to 18% in the 6OHDA- lesioned group (p<0.02; Fig. 4B). For VGLUT1-unlabeled terminals alone, there was no difference in the percentage of terminals making contact with spines vs. shafts (82%∶18%; Fig. 3B) between the control and 6OHDA-lesioned group (Fig. 4C, 4D). This contrasts with findings in the long-term MPTP-treated primate, in which there was no difference [7].

**Figure 5:**
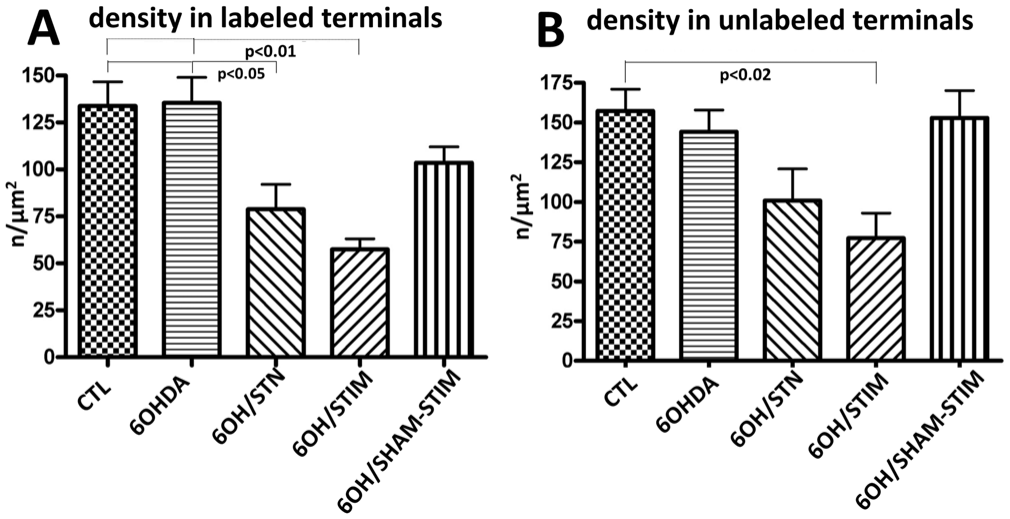
Density of immuno-gold labeled glutamate in asymmetric terminals. (A) In VGLUT1- labeled terminals there was no change in glutamate density with 6OHDA lesions. In 6OHDA-lesioned rats, STN lesions resulted in a decrease in glutamate particle density in VGLUT1-immunolabeled terminals (p<0.05), as did STN stimulation (p<0.01). 6OHDA-lesioned rats which underwent STN electrode placement alone did not show significant changes compared with 6OHDA alone. (B) Glutamate immuno-gold particle density in non-VGLUT1-immunolabeled terminals. There was no change in glutamate density in 6OHDA-lesioned rats in non-VGLUT1-immunolabeled, thalamostriatal terminals. There was a decrease in glutamate particle density with STN stimulation (p<0.02) as compared with controls, although only a trend to decrease (p<0.09) compared with 6OHDA-lesioned rats. The decrease with 6OHDA+STN lesions was not significant.

In 6OHDA-lesioned rats, there was no difference in the density of presynaptic glutamate immuno-gold-labeling (Fig. 2B) in either VGLUT1-labeled (Fig. 5A) or -unlabeled (Fig. 5B) terminals compared to controls.

### 6OHDA+STN lesions

For VGLUT1-labeled terminals alone, STN lesions (Fig. 1B) in 6OHDA- lesioned rats did not significantly change the percentages making synaptic contact onto a spine (Fig. 2C) or shaft (81%∶19%; Fig. 3C) compared with the 6-OHDA group (Fig. 4A, 4B). There was similarly no effect upon the percentages of non-VGLUT1-labeled terminals making contact onto spines or shafts compared with the 6OHDA-lesioned group (83%∶17%) (Fig. 4C, 4D).

Rats with 6OHDA+STN lesions showed a decrease in the density of glutamate immuno-gold-labeling (Fig. 2C) of 41% in VGLUT1-labeled terminals (p<0.05) as compared with 6OHDA-lesioned rats alone (Fig. 5A). The density of glutamate immuno-gold-labeling in unlabeled terminals was decreased by 30%, which was not significant (Fig. 5B).

### 6OHDA+STN stimulation

STN stimulation increased the percentage of VGLUT1-labeled terminals making contact on spines, and decreased the percentage of synapses upon shafts (95%∶5%; Fig. 3D) to values comparable to the control group (p = 0.05; Fig. 4A, 4B). There was no effect of electrode placement alone (sham stimulation) (Fig. 4E).

Rats with 6OHDA lesions+STN stimulation showed a decrease in nerve terminal glutamate immuno-gold-labeling (Fig. 2D) of 58% in VGLUT1-labeled (p<0.01; Fig. 5A) and of 47% in unlabeled terminals (p<0.02; Fig. 5B) as compared with controls. This was not seen with electrode placement alone (sham stimulation) (Fig. 2E, 5A, 5B).

### Post-synaptic glutamate labeling in dendritic spines

STN stimulation in 6OHDA-lesioned rats reduced the density of glutamate immuno-gold labeling within spines post-synaptic to VGLUT1-labeled terminals by 41%, which was non-significant (p = 0.17). However, for spines post-synaptic to non-VGLUT1-labeled terminals, the reduction was 47%, which was statistically significant (p = 0.05) (Table 2). The significance of this is unclear but may represent a metabolic response to increased synaptic activity, or a compensation for increased presynaptic glutamate release, and is possibly mediated by the glutamate transporter EAAC1. For all other groups, there was no difference in the density of glutamate immuno-gold labeling within spines compared to the control group regardless of whether the presynaptic nerve terminal was VGLUT1-labeled or -unlabeled.

**Table 2:** Density of immuno-gold-labeled glutamate particles in post-synaptic spines in which presynaptic terminals were VGLUT1-labeled or –unlabeled.

Group	VGLUT1-labeled	Non-VGLUT1-labeled
Control	142.8±24.1	149.8±12.1
6OHDA	130.1±35.2	138.4±14.0
6OHDA+STN lesion	103.8±56.2	94.6±15.3
6OHDA+STN stimulation	77.2±28.1	**72.9±17.1** *
6OHDA+sham stimulation	139.2±9.8	152.5±19.7

Mean ± standard deviation of number of particles/µm^2^.

* significantly different from 6OHDA alone p = 0.05.

## Discussion

### Comparison of STN stimulation and lesions

**Figure 6:**
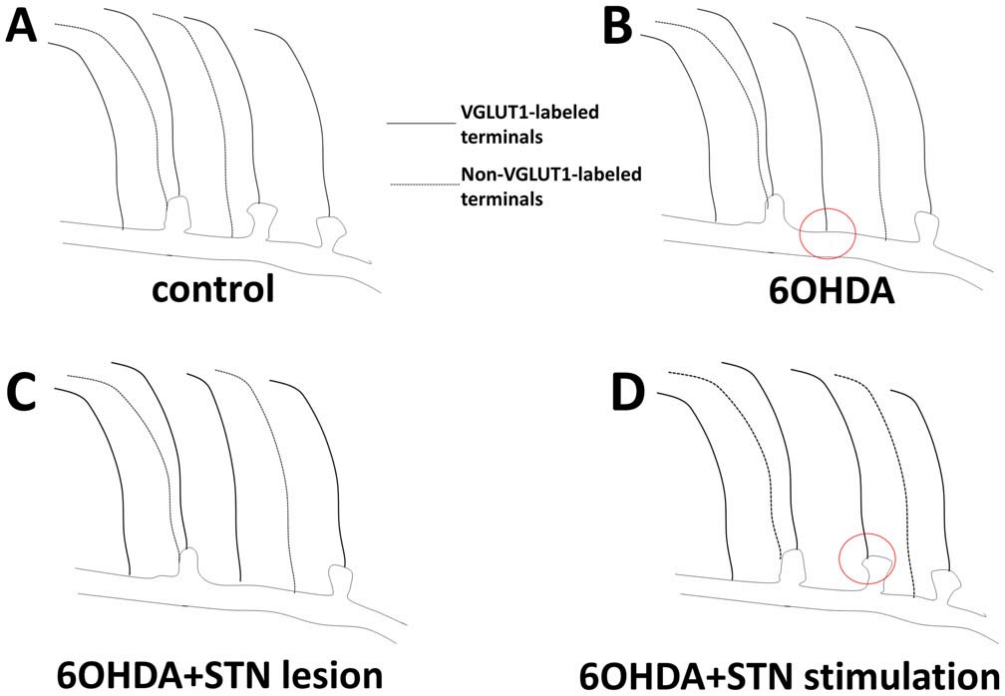
Summary of findings. Compared with control (A), in 6OHDA-lesioned rats, there were relatively more VGLUT-1 labeled terminals making synapses upon the shafts of dendrites (red circle; B). We hypothesise that this is due to fewer spines being available. There was no change in density of glutamate labeling. With STN lesions, there was decreased glutamate labeling in VGLUT1-labeled terminals, indicating increased release, indicated here by increased line thickness (C). With STN stimulation, there was decreased glutamate labeling, suggesting increased release in both types of terminals, and an increase in the proportion of VGLUT1-labeled terminals upon spines (red circle; D). We did not count spines, but postulate that there were more spines available for synapses due to plasticity. The relative numbers of VGLUT1-labeled and –unlabeled terminals and their locations are not accurate.

STN stimulation, but not STN lesions, returned the relative distribution of the locations of VGLUT1-labeled glutamatergic terminals to that seen in control rats (Fig. 6). This remodeling suggests that DBS may achieve its therapeutic effect, at least in part, by normalization of corticostriatal neurotransmission. As the number of spines is significantly reduced in the setting of dopamine depletion, both in rodents [29]–[33] and humans with Parkinson's disease [34], [35], it is possible that STN stimulation increases the number of spines available for synapses, maybe via a cortical mechanism [31], [36]. Quantitative stereological studies would be required to demonstrate this. Plasticity of glutamatergic terminals and post-synaptic spines has been reported in various conditions, with alterations in proteins involved in remodeling such as Arc, MEF-2 and Nurr77 [37]. Changes in morphology of striatal neurons with pruning of dendritic spines are noted in cell cultures depolarized by potassium within a few hours [37]. This suggests that rapid remodeling occurs in the striatum in response to changes in neuronal activity, and with changes in dopaminergic state, whether direct [38], [39] or indirect [40], [41].

Synapse location did not change with STN lesions for VGLUT1-unlabeled terminals, even when the density presynaptic glutamate immuno-gold labeling was decreased, possibly implying that this type of synaptic plasticity is of less importance for these (non-corticostriatal) afferents. An alternative explanation is that this finding indicates a more widespread mechanism of action of STN stimulation as compared with STN lesioning, potentially involving thalamostriatal pathways as well as corticostriatal. The finding of changes within one hour is consistent with the observation that the clinical response to DBS in PD is typically seen within minutes [42], however, it is certainly likely that distinct mechanisms come into play with sustained stimulation, which may also explain the difference found here between lesions and short term stimulation.

The mechanism by which DBS achieves its therapeutic effect is unknown. Although some studies of the effects of STN stimulation upon local neurons have reported inhibitory effects [43]–[46], others have not [47]–[49]. An inhibitory effect of stimulation upon STN neurons, with loss of drive of the GPi and disinhibition of the thalamus, would fit our data into the classic model of basal ganglia function. It is likely, however, that far more complex effects and circuitry are involved when the STN is stimulated, such as direct activation of nearby thalamostriatal fibres [50], or direct [51] or antidromic activation of the cortex [52], [53]. These mechanisms might result in stimulation of corticostriatal fibres and result in the remodeling we observed here. At a more system-wide level, DBS appears to normalize aberrant oscillations within the basal ganglia [54]. This widespread effect would not occur following an STN lesion.

Another hypothesis is that changes in striatal glutamate release and synaptic modeling may be secondary to alterations in striatal dopamine levels. We have previously demonstrated that STN stimulation affects striatal dopamine levels [16], possibly by an effect upon the remaining nigral dopamine neurons, and thus modulates striatal glutamate release via a presynaptic mechanism [55], [56].

### Effects of 6OHDA lesions

6OHDA lesions resulted in a small but significant shift of VGLUT1-labeled terminals from dendritic spines to shafts (Fig. 6), possibly due to a loss of spines [29]–[35]. There is debate as to whether spines are lost from both striatopallidal and striatonigral medium spiny neurons. Some studies have found evidence for spine loss only on D2 receptor-bearing striatopallidal neurons which comprise the indirect pathway [32]. The decrease in the percentage of VGLUT1-labeled terminals synapsing on spines could be explained if these terminals made contact with D2-containing neurons, however, this seems unlikely as recent data have shown that both types of striatal neurons are similarly innervated by both cortical (VGLUT1) and thalamic (VGLUT2) afferents [14]. In general, a global loss of spines across a large sample of striatal neurons is reported, rather than a bimodal distribution [29], [30], [33]–[35], [57] suggesting a general effect of dopamine loss upon both striatopallidal and striatonigral neurons. There is a similar decrease in the numbers of synapses and spines [27], thus the shift of synapses to shafts with dopamine depletion cannot be explained by the loss of spines available for synapses.

Following dopamine depletion, we found no differences in the density of glutamate immuno-gold labeling in either VGLUT1-labeled or -unlabeled terminals. As presynaptic depletion of neurotransmitter has been shown to correlate with increased synaptic release [4], [17], [58], [59], this result contrasts with evidence of increased striatal extracellular glutamate levels and glutamatergic activity by us [3], [4], and others [13], [60]–[64] and increased thalamostriatal activity [13], [65], at 2–4 weeks following 6OHDA lesions. Other groups, however, found glutamate levels to be unchanged [66]–[68].

Possible explanations for these differences may be the degrees of dopamine depletion, lesion sites, methodologies and time courses. However, it is possible that divergence in changes of presynaptic glutamate labeling and extracellular glutamate levels following 6OHDA lesions indicates that these are controlled by different mechanisms. Striatal extracellular glutamate levels reflect a complex balance between neuronal release and non-vesicular release related to glutamate transporters located upon both neurons and glia, and the cystine-glutamate antiporter [69], [70]. After dopamine depletion changes in glutamatergic neurotransmission within the striatum follow a complex, biphasic pattern within the first few weeks, possibly stabilizing by 3 months. Various markers of glutamatergic function have been examined at different time points, often with contradictory results, including the cystine-glutamate antiporter [71], and glial transporters [68], [72]–[75].

Vesicular neuronal glutamate transporters also follow a complex pattern after 6OHDA lesions. Studies of VGLUT1 found an increase in levels 3 weeks following dopamine depletion, and a subsequent decrease, while VGLUT2 levels initially decreased, then later normalized [76]. In studies of the MPTP-treated monkey, numbers of VGLUT1 transporters increased, but VGLUT2 was unchanged [7]. Depending upon their direction of change, these findings can be interpreted variously as being primarily responsible for changes in glutamate neurotransmission, or as compensatory mechanisms.

Striatal vesicular glutamate release is determined by a number of factors, including the activity of the afferent neurons, and local modulatory factors such as dopamine [55]. In general, the changes in presynaptic glutamate labeling found here most closely follow the changes we found in striatal dopamine levels in parallel experimental conditions [16], and support a close correlation between neuronal release of these two neurotransmitters [55], [56].

The difference in the current EM results from our previous report [4] may be due to methodological differences. Although we demonstrated >90% depletion of SNc TH-immunoreactivity, it is possible that medial forebrain bundle lesions [4] produced more complete dopamine depletion. A small percentage difference (from 90% loss of SNc neurons to 95–99% loss) has a large effect upon striatal metabolism [77], [78]. Although we saw comparable elevations in extracellular glutamate in our two publications [3], [4] the slightly lesser degree of dopamine depletion in the current study may not have been enough to affect vesicular glutamate release.

### Summary

6OHDA lesions resulted in a small but significant shift of VGLUT1-labeled terminals from dendritic spines to shafts. STN stimulation, but not lesions, returned the locations of these terminals to the proportions seen in control rats, suggesting that DBS may achieve its therapeutic effect, at least in part, by normalization of corticostriatal neurotransmission. This might be due to stimulation of corticostriatal pathways [51]–[53], and the relocation of terminals from shafts to spines, via mechanisms of synaptic plasticity, and hence increased efficacy of corticostriatal inputs [15].
